# eHealth Technology Competencies for Health Professionals Working in Home Care to Support Older Adults to Age in Place: Outcomes of a Two-Day Collaborative Workshop

**DOI:** 10.2196/med20.2711

**Published:** 2013-09-05

**Authors:** Ansam Barakat, Ryan D Woolrych, Andrew Sixsmith, William D Kearns, Helianthe SM Kort

**Affiliations:** ^1^Demand Driven Care, Research Centre for Innovations in HealthcareFaculty of HealthcareUniversity of Applied Sciences UtrechtUtrechtNetherlands; ^2^Gerontology Research CentreSimon Fraser UniversityVancouver, BCCanada; ^3^Department of Rehabilitation and Mental Health Counselling, University of South FloridaTampa, FLUnited States; ^4^Building Healthy Environments for Future UsersDepartment of the Built Environment, Building Physics and ServicesEindhoven University of Technology (TU/e)EindhovenNetherlands

**Keywords:** competencies, nurses, professionals, technology, CanMEDS, health care, eHealth, health information technologies, ambient assisted living, mobile health

## Abstract

**Background:**

The demand for care is increasing, whereas in the near future the number of people working in professional care will not match with the demand for care. eHealth technology can help to meet the growing demand for care. Despite the apparent positive effects of eHealth technology, there are still barriers to technology adoption related to the absence of a composite set of knowledge and skills among health care professionals regarding the use of eHealth technology.

**Objective:**

The objective of this paper is to discuss the competencies required by health care professionals working in home care, with eHealth technologies such as remote telecare and ambient assisted living (AAL), mobile health, and fall detection systems.

**Methods:**

A two-day collaborative workshop was undertaken with academics across multiple disciplines with experience in working on funded research regarding the application and development of technologies to support older people.

**Results:**

The findings revealed that health care professionals working in home care require a subset of composite skills as well as technology-specific competencies to develop the necessary aptitude in eHealth care. This paper argues that eHealth care technology skills must be instilled in health care professionals to ensure that technologies become integral components of future care delivery, especially to support older adults to age in place. Educating health care professionals with the necessary skill training in eHealth care will improve service delivery and optimise the eHealth care potential to reduce costs by improving efficiency. Moreover, embedding eHealth care competencies within training and education for health care professionals ensures that the benefits of new technologies are realized by casting them in the context of the larger system of care. These care improvements will potentially support the independent living of older persons at home.

**Conclusions:**

This paper describes the health care professionals’ competencies and requirements needed for the use of eHealth technologies to support elderly adults to age in place. In addition, this paper underscores the need for further discussion of the changing role of health care professionals working in home care within the context of emerging eHealth care technologies. The findings are of value to local and central government, health care professionals, service delivery organizations, and commissioners of care to use this paper as a framework to conduct and develop competencies for health care professionals working with eHealth technologies.

## Introduction

### Background

The world population continues to age [1], and the prevalence of chronic diseases is increasing [2], introducing complex societal challenges about how best to provide care to seniors. One in 5 workers will be employed in the health care sector by 2025, to meet the care demands of an aging population while supporting the independence, autonomy, and quality of life of older adults living at home [3,4]. To compensate for the anticipated shortfall in trained health care professionals, policymakers have advocated for the development and application of eHealth technologies as a potential tool to improve efficiencies in care [5]. While the application and deployment of eHealth technology has continued at a rapid pace, this has outstripped discussions on the skills and competencies that health care professionals are required to possess to successfully utilize the technology to support workplace practices.

eHealth involves the use of electronic communication and information technology to improve the access, efficiency, effectiveness, and quality of clinical and business processes utilized by health care organizations, health care professionals, and patients [6]. The term “eHealth” encompasses a broad range of technologies, including electronic/personal health records, telehealth, telecare, telemedicine, patient self-monitoring, ambient assisted living (AAL), and smart systems [6,7].

This paper focuses on health care skills and competencies required to utilize those technologies that support older adults aging in place: remote telecare and AAL, mobile health, and fall detection systems. This paper does not focus on health information technology (HIT) such as telemedicine or electronic health records. While there are differing occupations subsumed under the term “health care professional”, this paper refers to those who are future nurses (students) and current nurses. All types of nurses fall into this category including certified nurse assistants, licensed vocational/practical nurses, and registered nurses as key actors who will interface with eHealth in the future.

### Advantages of eHealth

Research indicates that eHealth technologies can yield substantial benefits for older people seeking to age-in-place by promoting independence and well-being while promoting efficiency and cost savings by reducing unnecessary hospital visits and delaying admission to long-term care [8,9]. Telehealth technologies, for example, facilitate remote patient consultations and monitoring of chronic health conditions at a distance [10]. Remote telecare technologies offer the potential to monitor and assist older adults with routine tasks and everyday home activities while enhancing their independence and autonomy [11]. As an example, simple automated reminders help and encourage older people to take medications or follow exercise programmes [12]. Other assistive and monitoring technologies, such as mobile health, environmental and body area networks in home, and health and activity monitoring, permit frequent serial patient observations of conditions or behaviors that assist caregivers by providing a more complete picture of patient status [13]. Technologies that facilitate the delivery of care in the home remove 3 barriers—stigma, access, and cost, which may prevent older people from presenting themselves to health care professionals [14,15].

eHealth interventions have the potential to alleviate the burden on health care professionals who have patients with complex care requirements or who currently manage high caseloads by providing the opportunity to monitor the condition of an older person remotely [16]. eHealth technologies have demonstrated success in allowing health care professionals to telemonitor blood pressure, pulse rate, and blood sugar levels, obviating the need for personal visits [17,18].

### Education and Training: Competencies

The advantages of eHealth technologies for health care professionals stem from a system that coordinates the collection, use and sharing of information to support health care delivery, known as health informatics [19]. Health informatics has developed rapidly in the last decade, becoming increasingly complex as technological advances and mechanisms for generating and sharing information have transformed clinical service delivery [20]. To ensure that these advances are translated into a service context, health care organizations must educate and train health care professionals in the latest tools and methods to accelerate the evolution of health care and affirm the acceptance of technology.

**Figure 1:**
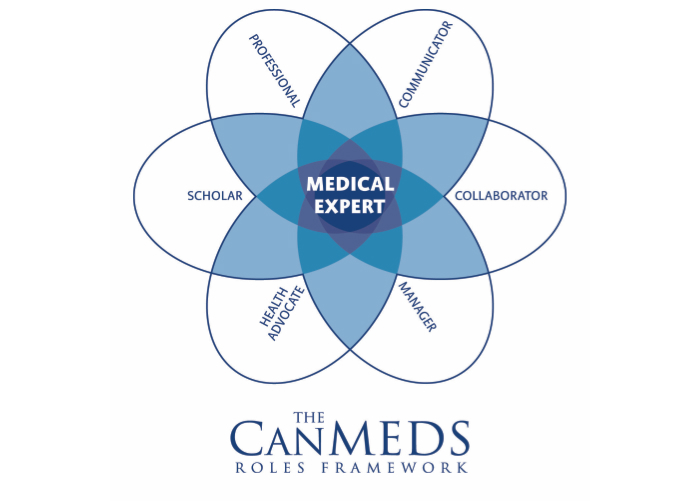
The CanMEDS framework.

The “Diffusion of Innovations” theory seeks to explain why, and how fast new ideas and technology spread through cultures [21]. The model introduces 4 key elements that influence the adoption of a new idea: (1) the innovation, (2) communication channels, (3) time, and (4) social system. Diffusion is the process by which an innovation is communicated through certain channels over time among the members of a social system. Rogers explained that individuals progress through 5 stages of adopting new technology: knowledge, persuasion, decision, implementation, and confirmation. The first step in the process of adaptation to and acceptance of technology is to enhance professionals’ knowledge about eHealth in their everyday work. Knowledge can be categorized according to minimum competencies that health care professionals are required to possess prior to applying eHealth technologies. The typology of skills adopted by the CanMEDS Physician Competency Framework presents 6 key roles for professionals when engaging in health care delivery, including advocate, communicator, collaborator, manager, scholar, and professional [22]. Figure 1 illustrates the central role of the medical expert and its interconnectedness with the other CanMEDS framework roles. This competency framework has been applied across different countries [23]; in the Netherlands, the CanMEDS framework is widely used in nursing education. The CanMEDS describes the composite roles required of health care professionals within generalized care delivery only and is not specific to eHealth. This highlights a potential disconnect between the increasing complexity of eHealth technology and the need to establish the composite skills required of health care professionals to make the best use of technology within a care context.

A systematic review indicated that end users’ HIT competencies and skills represent implementation barriers to eHealth [24]. In the Netherlands, competencies and skills have been identified as facilitators for the implementation of remote telecare to best support frontline nurses in the workplace context [25]. A description of competencies would ensure uniform quality of remote telecare service delivery with the potential to apply these skills to a broad range of health care decision-makers, including nurses, professions allied with medicine, social workers, health care managers, and caregivers.

### Early Research

In the Netherlands, van Merwijk [26] has described Information Communication Technology (ICT) as a fundamental component of remote nursing care delivery. However, eHealth training is not a core component for care professionals [27], although several studies have suggested that eHealth instruction should be integral to nursing education, with the responsibility for implementation falling on university educators, placement supervisors, and regulators [28,29].

There is a long history of development of competencies in the area of health informatics, nursing informatics, and (bio)medical informatics [30-34]. Peterson and Gerdin-Jelger [34] started in 1988 with the recommendations of the international medical informatics, which has been recently updated to accommodate the current developments on education in biomedical and health informatics [31]. Hasman and Albert [30] succeeded in suggesting a guideline for the European curricula in health informatics that apply to health care professionals and administrative staff. In the Netherlands, on-going work on competencies address nursing education and nursing informatics more specifically [35,36]. However, there is still little or no education for health care professionals in the use of technology to support older adults to age in place such as remote telecare, patient self-monitoring, and AAL. We contend that the current competencies must be adjusted to fully realize the benefits of eHealth.

Edirippulige et al [37] and Dattakumar et al [38] identified the absence of systematic nursing education and training in eHealth care as the reason for the emergence of under-skilled and ill-equipped eHealth practitioners. Other studies suggest that the skills gap has undermined confidence among nurses in eHealth technologies, with many stating they are not in a position to use these technologies effectively within the scope of their work [39,40].

### The Aim

In discussing the competencies of health care professionals working at home care organizations, 2 questions arise. First, which eHealth competencies are required for health care professionals to support older adults to age in place? Second, what is the nature of the supportive framework required to develop eHealth competencies? The goal of this paper is to present findings from a collaborative workshop tasked with exploring the basic eHealth competencies required of health care professionals working in home care, with eHealth technologies such as remote telecare, AAL, mobile health, and fall detection systems. In addition, we present a supportive framework that is required to establish these competencies in the field. This framework is an adapted framework to that used for HIT competencies.

## Methods

### Collaborative Workshops

In March 2012, the Department of Rehabilitation and Mental Health Counselling at the University of South Florida (USF) hosted an international group of academics in a set of collaborative workshops to discuss and explore eHealth competencies. Group discussion has been practiced extensively in participatory research to facilitate active dialog among individuals or groups to achieve the cross-fertilization of ideas [41]. Feldman [42] argued that engaging in collaborative dialog provided an opportunity to share and reflect upon experiences and to situate them within their broader context and meaning. Collaborative discussion allows for the transfer of knowledge, thoughts, and feelings about a topic of interest through a process of cooperative inquiry that enables new understandings to emerge (ie, a dialectical process). The workshops were funded as part of the Expanding eHealth Knowledge (iKOP) project, which investigates eHealth systems for their ability to support older adults living independently at home for as long as possible. The main research question of the iKOP project was “What criteria must eHealth fulfil to be understandable to professionals and to be used by older adults to reduce the burden of care and to reinforce independent living?” The workshops were designed to partly address the professional component of this question.

### Research Question

The aims of the collaborative workshops were as follows:

- The first aim is to share knowledge and expertise in the application of eHealth technologies with health care professionals through a process of collaborative learning.
- The second aim is to engage in collaborative discussion regarding the competencies required of health care professionals in the use of eHealth technologies.
- The third aim is to propose a set of skills and requirements for health care professionals to adopt eHealth technologies within their everyday working practices.

### Stakeholders

To develop the transdisciplinary dialog, 11 academics spanning the domains of biology, nursing, psychology, sociology, engineering, gerontology, and health management engaged in the collaborative workshops.

The participants were drawn from 3 academic institutions. First, academics from the USF included the chair and an associate professor of the Department of Rehabilitation and Mental Health and the interim dean of the College of Behavioral and Community Sciences (representing medical educators). Participants from USF also included a professor at the College of Nursing (representing medical educators and nurses), a research associate professor of the Department of Rehabilitation and Mental Health Counselling, and a courtesy professor at the School of Aging Studies (representing academics, professionals, and older adults). Second, academics from Utrecht University of Applied Sciences included a full professor and chair of the research group Demand Driven Care, Department of Health care, Research Centre Innovation Health Care, and a PhD candidate (representing older adults, professionals, home care settings, and academics). Third, academics from the Gerontology Research Centre at Simon Fraser University included a research fellow and a professor (representing professionals, home care settings, and academics).

In addition, two other organizations, the James A Haley Veterans Administration Hospital Centre of Excellence (JAHVA) and the Creative Action Limited Liability Company (CAL), were involved. From the JAHVA, a research health science specialist with interests in health disparities research and efficacy trials of health care technologies to inform translational research and system-wide implementation represented professionals (nurses) and academics. The Vice President of Research at CAL represented the interests of older adults, trainers, and software developers.

The participants had previous experience working with or evaluating the impact of eHealth technologies across a broad range of care contexts including home care and institutional care settings. A number of the participants have experience of developing technology with a focus on end user involvement and working alongside health care professionals to evaluate their experience of using eHealth technologies.

### Setting of the Workshops

The workshop consisted of two days of presentations and collaborative discussions to clarify the role of health care professionals in the application, development, and integration of eHealth technologies to support older adults to age in place. The presentations included a literature review on remote telecare competencies and requirements of health care professionals, feedback on the application and development of eHealth technologies in the homes of older people through funded research, and improvement of clinical research through the use of mobile technology. Three specific forms of eHealth technologies were addressed within the workshops: remote telecare and AAL, mobile health, and fall detection systems. These were the specific areas of expertise for the group of academics. Each presentation was followed by collaborative discussion in the group concerning the roles of health care professionals in using and delivering care through these different technologies and the specific competencies they might require.

At the end of each day of the workshop, a summary of the issues that were highlighted by all professionals was presented to the group for agreement. At the beginning of the second day of the workshop, a presentation was given about the key discussion points from the prior workshop day to refresh the participants’ memory. The competencies and requirements were selected by voting and ranking in importance while considering the diffusion of innovations theory by Everett Rogers and the CanMEDS Physician Competency Framework concerning how the competency or requirement aligns with new technology. The two-day workshop concluded with a presentation summarizing the workgroup’s recommended eHealth competencies and a discussion of future research directions. A number of participants kept written notes of the discussions, which were analysed thematically and presented in the results section.

## Results

### Necessary Competencies

During the workshop, eHealth products and possible necessary competencies were discussed. Participants agreed to structure the discussion as follows: (1) the requirements for basic ICT, proficiency, quantitative analysis, and interpretation skills, (2) communication skills, (3) support and guidance for the patient (both for care support, computer, and ICT use), (4) knowledge of best practices, and (5) legal requirements concerning patient privacy and confidentiality. Although health care professionals are required to possess a number of these skills within their current work role, the skills must be re-interpreted within the eHealth care context. Table 1 summarizes the competencies identified in the analysis of the workshop discussions.

### ICT Attitudes and Skills

The concept of eHealth is predicated upon sharing and communicating information through ICT technologies [6]. A desirable prerequisite for health care professionals is an abiding interest in the eHealth technology field. With little interest in eHealth technology, there will be scant enthusiasm to learn and adapt eHealth technologies for work roles or to apply learning obtained through formal training. Venkatesh et al [43] and Davis et al [44] showed in the technology acceptance model that intension to use is highly correlated to actual use. Moreover, an interest in eHealth care promotes sharing and learning within the workplace, establishing the foundation for positive cultural attitudes to develop toward the technology.

A crucial competency for health care professionals involves the basic skills for using technology and hardware, such as accessing the Internet or using a personal computer or mobile device [30,45]. Formal caregivers must have an aptitude for the devices used to collate, store, and display patient information in their new work routines [31]. Just as other researchers have found that basic skills in ICT are necessary [30,31], we agreed that professionals must be adept in using the software application to access patient information, to properly display and manipulate patient data and to ensure that information is interpreted correctly [30,31]. Developing competencies in using hardware and software are integral to ensuring the usability and acceptability of the device. Without these basic skills and aptitudes, care workers are likely to continue to rely on the traditional mechanisms of observation and monitoring, which they feel are more usable and familiar.

The key to developing trust in a specific system is for health care professionals to know the *how* and *why* of what specific technologies are designed to achieve within the homes of older people. Smith [46] has shown that for a system to be implemented successfully, it needs to yield benefits for the users. For example, developments in the area of smart and assistive technologies are increasingly reliant upon a suite of sensors and alarms to monitor the older person. During the workshop, we agreed that there is a need to translate the purpose of these sensors into easily understood, jargon-free language with the specific objective of understanding how a sensor collects, shares, and distributes information and why it is useful to the professional to know that information. For example, the use of door sensors helps to monitor patient activity, which informs the caregiver about how many bathroom visits the person has performed. This is important for understanding how technologies can support the health care professional’s everyday working practices. Another key area that can undermine the acceptance of eHealth technologies is the reliability of the technology [47]. Telephone technology operates with “Five 9s” reliability; in other words, it is available 99.999% of the time, with infrequent outages resulting from events such as severe weather [48]. Computers and Internet-based technologies are somewhat less reliable, and Internet protocols are termed “best effort” service delivery functions. Best effort service is, by definition, not perfect, nor should it expected to be. Information packets can be dropped or delayed, resulting in an incomplete delivery of eHealth services [49]. Mobile devices that rely upon the eHealth professional to properly charge the battery and maintain the Internet service provider account may find that access in a given region is not accessible due to poor network coverage or a discharged battery, meaning that the eHealth technology does not function when it should. The competent eHealth service provider must be cognizant that the failure of technology is not an infrequent event and that its successful resolution hinges upon the professional exercising a combination of tact, grace under fire, and patience. Technology failures undermine the confidence of the patient and the professional who uses them. With the expansion of mobile technologies into the everyday lives of citizens, we find that the general population has become somewhat more tolerant of service interruptions due to computer viruses, lack of cellular telephone service, and incorrectly configured networks [50]. Nevertheless, the willingness to use technology decreases if it is perceived as being more trouble than it is worth.

### Interpretation and Analysis of eHealth Solutions and Data

It is necessary to ensure that health care professionals have the skills to interpret patient information gathered with eHealth technology. The presentation of information clearly, concisely, and in an interpretable way is a technological requirement, but eHealth care may require a different instrument to interpret information for health care professionals so that it makes sense in the context in which it was generated. The type of data generated through e-technologies may vary from longitudinal monitoring data to more immediate observations of a patient’s condition, and the health care professional must competently interpret the new data. Another skill required of the health care professional is translating the data into meaningful information for effective clinical decision making by combining the data with the professional’s knowledge of the patient’s health condition and the health care domain to derive the most appropriate, least burdensome, and most cost-effective intervention.

### Support and Guidance

If eHealth is to produce efficiency savings through economies of scale, then care will need to be increasingly delivered remotely, through mobile consultations or networked care delivery that obviates the need for direct contact. Given that the caregiver will not have face-to-face contact with the older person, good support and clear guidance to the patient will be important to ensure that health problems can be effectively diagnosed and treated at a distance [50].

The professional caregiver will be required to provide on-going support and guidance in the use of the technology to the patient. For example, where remote self-monitoring provides patients with access to their own data, health care professionals must educate users in the functionality of the system and in interpreting the readings so that they feel empowered in the decision-making process. In the expansion of e-telecare, the role of the health care professional as an educator and facilitator for the patient is important if eHealth technology is to become accepted in the home environment. The empowering role of the health care professional has been observed in the development of the expert patient programme in the United Kingdom and within the broader role of case management [51]. Evaluations of AAL technologies have highlighted the importance of community nurses in facilitating the benefits of technology when making care-related decisions. Here, community nurses possess tacit knowledge of the end user and their own expert knowledge in health care delivery and clinical decision making, which, when combined with data generated through assistive technology, can improve the usefulness and usability of eHealth care interventions [52].

### Communication Skills

#### eHealth Care

eHealth care has the potential to change the dynamics of care and to bring about changes to the types and intensity of verbal and nonverbal communication in the care dyad. In the following sections, adjustment and modification of the communication between two caregivers or between the caregiver and the client is described.

#### Communication Skills Between Health Care Professionals and Clients

Effective communication skills are a current requirement for health care professionals, but eHealth care technologies have changed the traditional modes of interaction between caregivers and clients. Different technologies present alternative ways for health care professionals to project themselves into the care setting, such as by email, telephone, mobile devices, or teleconferencing. In doing so, health care professionals must be aware of how technology nuances their communications to deliver the type of care and person-centred support the end user requires.

The health care professional must ensure clear and transparent communication between the professional and the user. Voice intonation, listening skills, and clarity of two-way communication are important when delivering messages via technology because face-to-face prompts and supports are absent. As an example, remote telecare health care professionals communicate *synchronously* via an audio/video connection. Specific competencies are required for remote telecare, including presenting a professional appearance, sensitivity to maintaining eye contact with the client, adopting an engaging facial expression, and having a well-developed ability to recognize changes in client behaviors or environmental surroundings via the telecommunications link. These aspects may be conveyed differently through teleconferencing. It is worth noting that colors may not be correctly translated by all video devices in use in telecare situations and that high ambient noise levels may cause the professional to miss subtle cues that might be present in the individual’s voice. These environmental considerations are classed broadly in the telecommunications as “production values” and subtly affect the recipient of the communication. When completing observations or encouraging patients to undertake a task, the professional cannot support the client by physically guiding them through the process but instead need to rely primarily upon verbal communication and nonverbal gestures, such as nodding or facial expressions to express satisfaction. The communication must be individually tailored because some persons require more frequent contact depending on their cognitive capacities and their specific social and emotional support needs.

#### Communication Skills Between Health Care Professionals

The health care professional may be required to engage other health care professionals when making care-related decisions and must ensure that patient health information is shared responsibly [53-56]. eHealth technologies should be set up to facilitate sharing information between and across organizations, but professionals need to engage in regular communication to facilitate joint working with other stakeholders who are responsible for delivering care to the older person, such as informal caregivers, health care delivery organizations, and community and voluntary groups. Therefore, an adjusted version of the health level 7 standards and the ISO 13606 could be used to apply to specific eHealth technologies [53-56]. However, health care professionals must be aware of the far-ranging implications of eHealth solutions across the broader integrated spectrum of care.

### Privacy and Confidentiality

The sharing of patient information across an integrated eHealth system raises questions about patient privacy and confidentiality [57]. Health care professionals must be aware of the specific ways in which eHealth technologies have the potential to compromise the privacy and confidentiality of the patient and of the rights of the patient to know how these data are shared and viewed.

Even though there are standards for data and information sharing [58,59], eHealth care changes the ways in which health care professionals observe, view, and share information, leading to unique requirements for how patient data are retained and kept secure from others when using mobile devices and online tools. Moreover, if eHealth draws upon multiple professionals from various service providers, there is a need to be aware of who is allowed access to what information. Unfettered access to patient information is neither desirable nor, in some countries, legal, but there is a need to ensure the effective sharing of information across service providers while taking into account (inter)national privacy law and legislation rules.

**Table 1:** Skills and competencies of health care professionals.

Theme	Competencies	Requirements	Skills
ICT attitudes and skills	Competent in the use of necessary telehealth technologies and software and adopts a positive attitude toward their use in the workplace	Have an abiding interest in the eHealth technology field	Know specific skill sets in eHealth technologies being applied
			Have basic skills for using technology and hardware, such as accessing the Internet or using a personal computer or mobile device
			Have an aptitude for the devices used to collate, store, and display client information
			Know and be able to translate the benefits of eHealth technologies to end users
Interpretation and analysis of eHealth data	Competent in interpreting end user data and applying these data to effective clinical decision making	Knowledge of the client’s health condition and the health care domain	Ability to interpret output data generated by eHealth care technologies
			Translate the data effectively within the context of the client with a positive outcome
Support and guidance	Ability to provide on-going support and guidance to end users to increase the acceptability of eHealth technologies	Possess tacit knowledge of the end user and their own expert knowledge in health care delivery and clinical decision making	Educate end users in the operation and functionality of the technology
			Ability to diagnose and treat effectively at a distance
			Effectively combine clinical knowledge with eHealth data in decision making
Communication skills	To communicate effectively with both end users and formal care providers	Have general communication skills	Ability to have clear and transparent communication between the professional and user, such as voice intonation, listening skills, and clarity of two-way communication
		Be aware of the far-ranging implications of eHealth solutions across the broader integrated spectrum of care	Ability to interpret verbal and nonverbal cues, such as nodding or facial expressions, in interaction with end users
			Facilitate information sharing and transferral across formal care providers
Privacy and confidentiality	To maintain the privacy and confidentiality of the end user	Be aware of the privacy and confidentiality rules of data exchange	Need to secure all personal health data for the patient
			Ensure that information transferral and exchange takes place within a secure platform; apply the concept of least privileged access to other practitioners sharing confidential information

## Discussion

### Principal Findings

In this paper, we described the competencies and requirements needed by health care professionals for the use of eHealth technologies to support older adults to age in place. The results of this paper are in good agreement with those of Kulikowski et al [32], IMIA group [31], Hasman and Albert [30], Goossen et al [35,36], and Ayres [45] with regard to general competencies (ie, computer skills and informatics knowledge). We expanded those competencies to extend to the more specific use of technologies such as remote telecare and AAL, mobile health, and fall detection systems. Moreover, this paper proposes a supportive framework required to establish these competencies, other than the HIT competencies, in the field of professionals working in health care to support older adults to age in place. In addition to the described competencies, this paper highlights a number of barriers and facilitators to applying and developing these competencies within the health care profession beyond ensuring that they are a fundamental aspect of training and education [21-24]. However, there are still a number of strengths, weaknesses, opportunities, and threats in the internal and external work environment that must be mapped for the adoption of eHealth technologies.

Strengths include those skills that health care professionals currently possess when delivering care to clients or patients and that can be capitalized upon in the integration of eHealth care, including the ability to analyse and interpret data from existing monitoring mechanisms, tacit knowledge of the client and the home environment, and clear and transparent verbal and nonverbal communication skills to establish trust and reciprocity with clients. In the Netherlands, based on the CanMEDS systematic the seven following workplace skills for nurses are described: clinical performance, communication, cooperation, organization, social performance, knowledge and science, and professionalism [26]. With this in mind, the theme “ICT attitudes and skills” as described in this paper can be seen as knowledge and science skills, whereas skills described under the theme “interpretation and analysis of eHealth data and support and guidance” might be seen as clinical or social performance. Further, the themes “communication and privacy, and confidentiality” can be seen as communication and professionalism skills, respectively. Moreover, communication skills have always been important in establishing rapport and trust with patients, and these are equally important within the context of eHealth care. These strengths can ensure that the benefits of eHealth technologies can be maximized to bring about improvements to the delivery of care (positive outcomes) and to realize efficiencies for the health care professional (better management of caseloads). All of these skills are integral to ensuring that eHealth technologies become widely adopted within the home environment. Existing skills, such as the ability to review paper-based patient case notes, can be transferred to eHealth solutions by utilizing different instruments to collect and display that information. A tacit understanding of the client enables eHealth data to be interpreted within the context in which they were generated, ensuring that interventions are appropriate. This implies organizational skills. Ensuring appropriate interventions has already been achieved for health information technology such as ICT and electronic public records, however not yet realized for remote telecare and assistive living technology.

In recognizing the strengths that health care professionals possess, there is also a need to recognize the weaknesses that must be addressed. eHealth technologies will bring about changes to the health care profession. The very notion of eHealth supporting or replacing tasks that the health care professional traditionally undertakes may evoke hostility within the profession slowing widespread adoption [25]. Interventions need to be integrated in an appropriate and sensitive manner and adapted to the existing workplace practices and workflow of the health care professional [25]. Moreover, although the benefits of eHealth care technologies have been documented, there is a dearth of large-scale evaluation studies of their long-term impact. This lack of an evidence base undercuts the argument that eHealth care technologies are necessary for the health care professional.

### Opportunities

Despite these weaknesses, there are a number of opportunities available to ensure that eHealth care becomes a fundamental part of care delivery to support aging-in-place, and these opportunities require a number of changes at the organizational level. Following Rogers’ model for adopting new technology, we argue that first, although knowledge can be derived through the training and education of health care professionals, there must be systematic mechanisms in place for reviewing training needs and requirements within the context of emerging and changing technologies. Second, for persuasion, organizational commitment is needed to ensure the development of a culture of working with eHealth technologies, encouraging health care professionals within organizations to share their experiences with other professionals when using technologies. This will help in identifying and working through barriers while exploring the unanticipated benefits of using technology within the profession. Third, to support organizations in their decision of using eHealth, organizations must demonstrate that eHealth solutions are an essential part of delivering care in the future and must instill this within the cultural ethos of the organization, which challenges the traditional approaches to delivering care. Fourth, for a successful implementation, the benefits of using eHealth solutions need to be mapped and translated to caregivers. If eHealth technologies can enable health care professionals to accomplish their everyday tasks more efficiently, and if professionals can see the benefits in terms of assisting them in their roles, then professionals are more likely to give their confirmation to use eHealth technology within their everyday working practices.

### Threats

A number of existing threats to the successful integration of eHealth care prevent the strengths and opportunities of eHealth care technologies from being realized. These include the conservatism inherent in the care delivery system, existing ways of commissioning care, traditional approaches to care delivery, and a lack of flexibility within health care systems to accommodate innovation and change. Care delivery is complex and involves multiple providers at the local level, the architecture of which differs across jurisdictions but involves a number of health care professionals. eHealth technologies must be sufficiently flexible to facilitate integration across jurisdictions; otherwise, health care professionals will be asked to work within complex circumstances. Ultimately, eHealth technology must be seen as part of the broader cultural system of delivering care to ensure that eHealth care becomes part of an integrated system of delivery.

An additional threat to the integration of eHealth care is the low level of funding for eHealth-based care delivery [60] and the lack of standardization in the field of eHealth. The ISO 13131, Health Informatics-Quality criteria for services and systems for telehealth, focuses on establishing standards for eHealth, allowing payment schemes to be defined [61].

### Conclusion

Advances in eHealth technology have the potential to bring about efficiency savings in terms of delivering care to older people and to support self-management by older adults. To facilitate this scenario, there is a need to ensure that the pace of technology development is reflected in the abilities and skills of health care professionals working in home care organizations. In the eHealth world, professionals are required to collate, share, and manage multiple forms of information and to interact with different types of technology in their everyday working practices. This requires that the role of the health care professional be revisited to examine the existing skills gap and to identify professional development opportunities and educational needs. Technologists and engineers have been concerned with developing technological solutions to health and social care problems, but much of the research so far has been limited in terms of real-world products and services [61,62]. A number of barriers to effective implementation exist. In particular, there has been insufficient attention paid to the ways in which technology can be integrated into the working practices and workflow of care professionals [25]. Further work must be undertaken to examine the experiences of health care professionals when using the broad range of technologies on the market and to remove the barriers and establish facilitators to the realization of these technologies within an organizational context. The findings presented in this paper are exploratory and are limited in that they draw upon the opinions of academics as opposed to stakeholders involved in the commissioning of care. Future work should consider the perspectives of a broad range of stakeholders and actors involved in designing and commissioning technologies that change the way health care professionals remotely access patient information to support them to live independently at home.

## Abbreviations

AAL: ambient assisted living
CAL: Creative Action Limited Liability Company
HIT: health information technology
ICT: Information Communication Technology
iKOP: Expanding eHealth Knowledge
JAHVA: James A Haley Veterans Administration Hospital
USF: University of South Florida
